# Stress‐induced body weight loss and improvements in cardiometabolic risk factors do not translate to improved myocardial ischemic tolerance in western diet‐fed mice

**DOI:** 10.14814/phy2.15170

**Published:** 2022-01-25

**Authors:** Kyle Hatton‐Jones, Amanda J. Cox, Jason N. Peart, John P. Headrick, Eugene F. du Toit

**Affiliations:** ^1^ School of Pharmacy and Medical Science Griffith University Southport Queensland Australia

**Keywords:** anxiety‐like behavior, cardiometabolic risk, chronic stress, myocardial ischemia, restraint stress: diet, western diet

## Abstract

Although both diet‐induced obesity and psychological stress are recognized as significant independent contributors to cardiometabolic and behavioral disorders, our understanding of how these two disorders interact and influence cardiometabolic risk and myocardial ischemic tolerance is limited. The aim of this study was to assess the combined effects of an obesogenic diet and psychological stress on cardiometabolic risk factors (body weight, dyslipidemia, insulin sensitivity) and postischemic cardiovascular outcomes. C57Bl/6J mice (*n* = 48) were subject to a combination of 22 weeks of western diet (WD) feeding and chronic restraint stress (CRS) for the last 4 weeks. Metabolic and behavioral changes were assessed using glucose tolerance tests and open field tests (OFTs), respectively. After 22 weeks, cardiac function and ischemic tolerance were assessed in Langendorff perfused hearts. WD feeding increased body weight and worsened blood lipids and insulin sensitivity. WD‐fed mice also exhibited reduced exploratory behavior within the OFT. CRS reduced body weight and increased locomotion in both dietary groups and had differential effects on fasting glucose metabolism in the two dietary groups while not impacting non‐fasting insulin. Although the WD only marginally reduced reperfusion left ventricular developed pressure recovery, CRS worsened reperfusion diastolic dysfunction in both dietary groups. Interestingly, despite WD+CRS animals exhibiting improved cardiometabolic parameters compared to the WD group, these changes did not translate to marked improvements to postischemic cardiac outcomes. In conclusion, in this study, combined WD feeding and CRS did not act synergistically to worsen cardiometabolic risk factors but instead improved them. Despite these cardiometabolic improvements, WD+CRS increased reperfusion end diastolic pressure which may be indicative of worsened ischemia/reperfusion injury.

## Graphical abstract

Although both diet‐induced obesity and psychological stress are recognized as significant independent contributors to cardiometabolic and behavioral disorders, our understanding of how these two disorders interact and influence cardiometabolic risk and myocardial ischemic tolerance is limited. The aim of this study was to assess the combined effects of diet and psychological stress on cardiometabolic risk factors and postischemic cardiovascular outcomes.

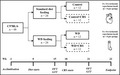

**New findings**

- What is the central question of this study?

Both diet‐induced obesity and chronic stress are associated with metabolic, behavioral, and cardiovascular abnormalities; however, our understanding on how these two risk factors interact to influence ischemic tolerance and postischemic cardiac outcomes is limited.

- What is the main findings and its importance?

Using C57Bl/6J mice subject to both a chronic western diet and chronic restraint stress, we show improvements to body weight and glucose tolerance in obese mice subject to stress. These metabolic improvements did however not translate into improved myocardial ischemic tolerance but rather worsened select functional outcomes. These outcomes suggest stress may cause a dissociation between traditional cardiometabolic risk factors thought to decrease myocardial ischemic tolerance and post‐myocardial infarct outcomes via yet unresolved mechanisms.

## 1 INTRODUCTION

In recent decades, the incidence of obesity and depression has increased to the extent that they now represent two of the most pressing and costly chronic health conditions globally. The associated burden of disease is significant for both the individual and the health care system, particularly given the recognized association between these disorders and the risk for additional comorbidities such as coronary heart disease, type‐2 diabetes, and neurocognitive decline that all contribute to premature mortality (Abdelaal et al., 2017; Andre et al., 2014; Gheshlagh et al., 2016; Gilman et al., 2017). Indeed, recent studies link stress to exacerbated metabolic syndrome in obesity and posits that the two conditions may act synergistically to promote disease (Chandola et al., 2006; Janczura et al., 2015; Rosengren et al., 2004). Importantly, both obesity and chronic psychological stress increase the risk for myocardial infarction (Arnold et al., 2012; Jackson et al., 2018; Yusuf et al., 2005). Considering the increasing prevalence of individuals with multimorbidity, and an established bidirectional relationship between obesity and depression, our understanding of how these two chronic conditions interact to impact myocardial ischemic outcomes remains limited (Harrison et al., 2017; Mannan et al., 2016).

Experimental evidence suggests that dyslipidemia, insulin resistance, inflammation, and oxidative stress are key mediators of ischemic intolerance in obesity (Liu & Lloyd, 2013; Morel et al., 2003; Thakker et al., 2006; Toit et al., 2008). Data from human studies support a role for psychological stress in the promotion of weight gain, insulin resistance, and inflammation, and thus one could posit that chronic stress could synergistically worsen ischemic intolerance in obesity (Cuevas et al., 2019; Harris et al., 2017; Miller et al., 2008). Studies assessing the combined effects of diet‐induced obesity (DIO) and psychological stressors in animal models yield mixed results in terms of metabolic outcomes. This includes reports of both adverse and beneficial effects on cardiometabolic risk factors such as body weight, glycemic handling, and circulating insulin (Aslani et al., 2015; Finger et al., 2012; Fu et al., 2009; Kai et al., 2000; Sousa Rodrigues et al., 2017). Few studies have assessed interactions between DIO and psychological stress in governing cardiovascular phenotype and disease pathogenesis (Agrimi et al., 2019; Crestani, 2016; Du Toit et al., 2020). Thus, the relationship between metabolic homeostasis and cardiovascular outcomes within the context of chronic stress and DIO remains enigmatic.

In our previous work, we demonstrated a synergistic worsening of metabolic homeostasis with a concurrent reduction in myocardial ischemic tolerance in mice subject to 12 weeks of western diet (WD) feeding and chronic restraint stress (CRS). However, considering the mixed findings in the field regarding the metabolic outcomes in models combining diet and stress, we aimed to investigate the effects of a more robust model of CRS on our established model of DIO. We hypothesized that the combination of a WD and CRS would act additively to worsen metabolism, behavioral markers of anxiety, and myocardial susceptibility to ischemic/reperfusion (I/R) injury as per our previous findings. Contrary to the findings of our previous study, we found evidence of improvements to cardiometabolic risk factors (body weight, lipids, and insulin sensitivity) in the WD+CRS groups in the current study utilizing a more severe CRS intervention. These changes were however not accompanied by similar improvements to postischemic cardiac outcomes, suggesting a disconnection between metabolism and cardiovascular outcomes in certain models of stress.

## 2 METHODS

### 2.1 Animals and experimental procedure

**FIGURE 1:**
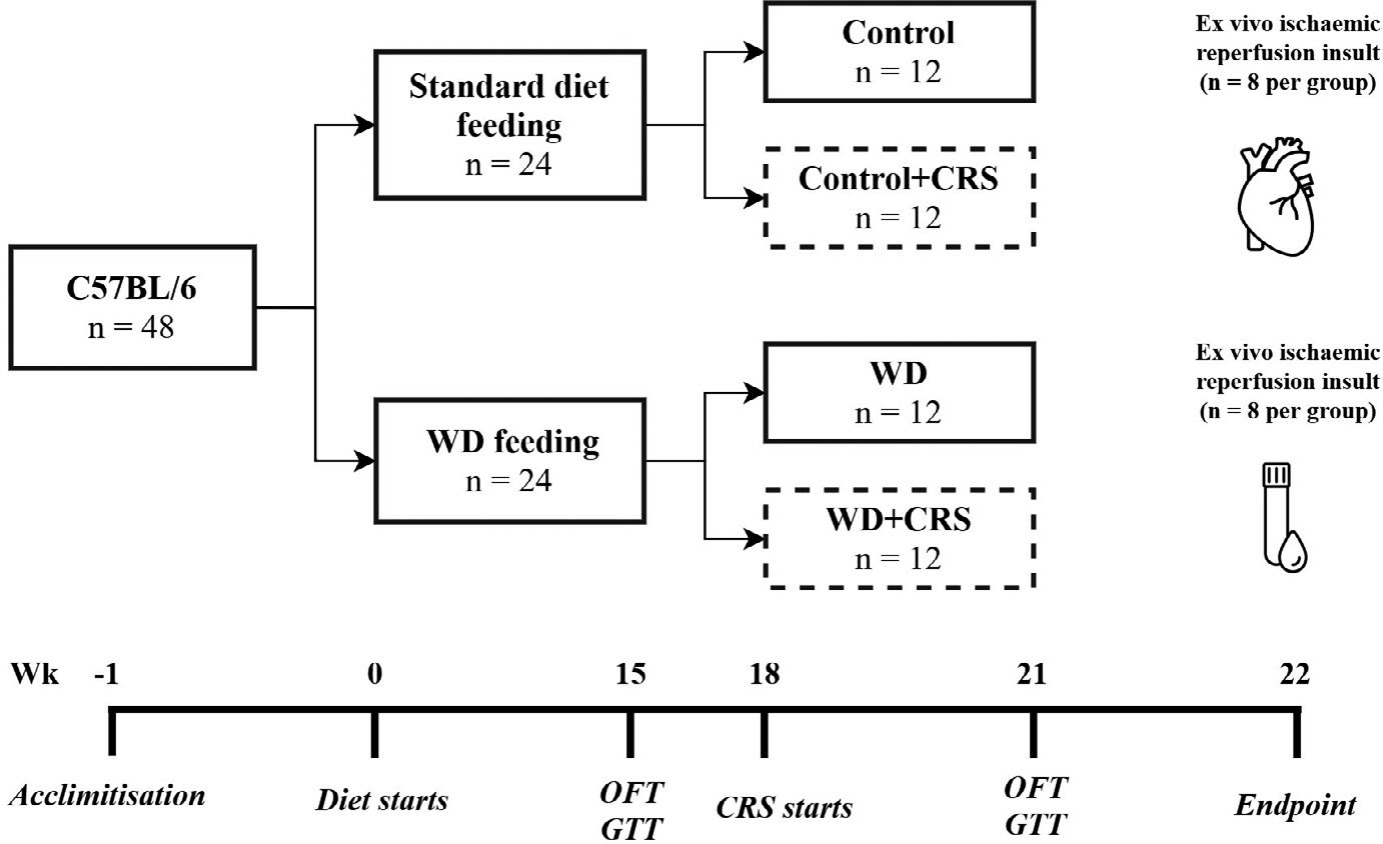
Schematic of 22‐week study design. At endpoint, blood was collected from the thoracic cavity for assessment of circulating metabolic markers. In a subset of mice (*n* = 8 per group), hearts were excised and lung onto a Langendorff perfusion system to assess myocardial tolerance to ischemic/reperfusion injury. CRS, chronic restraint stress; GTT, glucose tolerance test; OFT, open field test; WD, western diet

Forty‐eight C57Bl/6J male mice at 8 weeks of age were purchased from the Animal Resource Centre, Western Australia and were housed in an environmentally controlled animal care facility for 1 week. After acclimatization, the mice were housed in cages of six at 22°C under a 12‐h‐light/dark cycle with free access to food and water. Mice were randomly divided into two dietary groups and were provided with either a control or WD chow ad libitum for a period of 22 weeks (Figure 1). The Control group (*n* = 24) were maintained on a standard rodent chow (Specialty Feeds): The WD group (*n* = 24) was fed a diet of standard rodent chow supplemented with refined sugar, animal fat, and condensed milk in order to promote DIO as per our previous work. (Du Toit et al., 2020). The WD was modeled to equate to human WD (49% from carbohydrate, 35% from fat, and 16% from protein) in the US population (NHANES, 2013–2014). Content and nutritional details of the control and WD diet can be found in Table 1. After 18‐weeks, mice from each dietary group (*n* = 12) were randomly allocated to CRS for the final 29 days of the study. The remaining mice from each dietary group (*n* = 12 each) were handled for an additional 5 min but not subjected to the stressor. The four experimental groups were: (1) Control; (2) Control+CRS; (3) WD; and (4) WD+CRS. Prior to (Week 15) and after the 4‐week CRS period (Week 21) mice were subject to an open field test (OFT) and a glucose tolerance test (GTT) to assess diet and stress‐induced changes to behavior and glucose handling.

**TABLE 1:** Dietary content and ingredient breakdown

	Source	Macronutrient breakdown (%kcal)		
		Carbohydrates	Fats	Protein
Standard rodent chow	Speciality feeds; meat free rat and mouse diet (wheat, barley, lupins, soya meal, fish meal, mixed vegetable oils)	64.1	14.4	21
Western diet	Standard rodent chow (800 g) supplemented with 125 g of animal fat, 140 g of refined sugar, and 794 g of condensed milk	56.7	30.2	11.3

At the end of the experimental period (Week 22), mice were anesthetized between 08:00 and 10:00 h using sodium pentobarbital (60 mg kg^−1^ i.p.) and euthanized by removal of the heart and subsequent exsanguination. Non‐fasted blood glucose was determined using tail blood and an Accu‐check II glucometer (Roche Diagnostic). In a subset of mice from each group (*n* = 8/group), hearts were removed and Langendorff perfused to assess cardiac function and responses to ischemic insult. Blood was drawn immediately from the thoracic cavity after heart excision for further metabolic and corticosterone analysis.

### 2.2 Chronic restraint stressor

For mice allocated to the psychological stressor groups, CRS was used as described previously to induce chronic mild stress (Bowers et al., 2008; Chiba et al., 2012). Mice were restrained by placing individual animals in acrylic chambers for 1 h day^−1^ for 3 weeks and 5 days. All mice were weighed daily during the 29 days of CRS and monitored daily for behavioral abnormalities or signs of distress throughout the final 4 weeks. The stressor was not applied on days when behavior testing or GTT analyses were performed, and was terminated 2 days prior to animal euthanasia, blood collection, and heart perfusions.

### 2.3 Body weight and GTTs

Mice were weighed weekly during feeding and daily during the CRS period using an electronic laboratory scale. Glucose tolerance was assessed prior to and following the 4‐week CRS period using a GTT. Mice were fasted for 4 h prior to GTTs. Blood was obtained via tail tipping and blood glucose determined using an Accu‐check II glucometer (Roche Diagnostic). A 20% (2 g kg^−1^) glucose bolus was administered via intraperitoneal injection and blood glucose levels recorded at 15, 30, 60, 120, 180 min post injection. Total area under the glucose curve (AUC) was calculated using all time‐points as a measure of glucose tolerance (GTT‐AUC).

### 2.4 Open field test

Locomotor activity and exploratory behavior were assessed in all mice prior to and following the 4‐week CRS period, during a 30‐min period in an OFT arena. The OFT is a validated tool for the assessment of anxiety‐like behavior in a foreign environment (Katz et al., 1981; Sestakova et al., 2013) and was used to determine the impact of the WD and CRS intervention on anxiety‐like indices. The arena (70 × 70 × 56 cm) floor was subdivided into a 4 × 4 marked grid and a center square, with an overhead digital camera recording all activity. Briefly, mice were transported to the testing room and habituated to the arena room for 30 min prior to testing. The arena was cleaned with 80% ethanol (w/v) between each test. The mouse was placed within the center square of the arena and the test began when the mouse left the center square. Videos were analyzed using a custom animal tracking software (Hatton‐Jones et al., 2021). The primary variables assessed using tracking software included horizontal locomotive activity (distance in cm), and exploratory behavior via center square entries (CSE) and center square duration (CSD).

### 2.5 Cardiac function and ischemic tolerance

After anesthesia, hearts were excised into ice‐cold perfusion fluid, the aorta was cannulated and hearts mounted on a Langendorff perfusion system (Reichelt, Willems, Hack, Peart & Headrick, 2009). Hearts were perfused with modified Krebs–Henseleit buffer (119 mM NaCl, 11 mM glucose, 22 mM NaHCO3, 4.7 mM KCl, 1.2 mM MgCl2, 1.2 mM KH2PO4, 1.2 mM EDTA, 0.5 mM, and 2.5 mM CaCl2) bubbled with 95% O2/5% CO2 at 37°C (pH 7.4) and were immersed in perfusion fluid a 5‐ml water jacketed organ bath (also set to 37°C).

To measure contractile function a fluid‐filled balloon was inserted into the left ventricle and inflated to an end‐diastolic pressure (EDP) of 5 mmHg during stabilization. An ultrasonic flow‐probe in the aortic perfusion line, connected to a T206 flow meter, provided a constant measure of coronary flow rate (Transonic Systems Inc.). Hearts were stabilized for 20 min and then switched to ventricular pacing at 7 Hz (SD9 stimulator; Grass Instruments) for 10 min prior to ischemia‐reperfusion. Global normothermic ischemia was induced for 25 min, followed by 45 min of aerobic reperfusion. Time spent in ischemia was chosen based on previous work done by our lab and others (Du Toit et al., 2020; Wang et al., 2001). Peak systolic and EDP, heart rate, flow, and + and −*dP*/*dt* were continuously monitored at 1 kHz using an eight channel MacLab system (ADInstruments Pty Ltd.) connected to an Apple iMac computer. Thermal probes connected to a three‐channel Physitemp TH‐8 digital thermometer monitored the temperatures of the perfusate and water bath (Physitemp Instruments Inc.; Reichelt, Willems, Hack, Peart & Headrick, 2009).

### 2.6 Blood serum analyses

To assess the effects of the WD and psychological stress on cardiometabolic risk factors, non‐fasted blood was collected at sacrifice to assess circulating levels glucose, triglycerides, insulin, and corticosterone. Glucose was determined immediately in whole blood using an Accu‐check II glucometer (Roche Diagnostic). Serum samples were stored at −80°C for subsequent metabolic analyses. Serum triglycerides were assayed using a triglyceride quantification colorimetric kit (Biovision Inc.), serum insulin using an ultra‐sensitive mouse insulin ELISA kit (Crystal Chem), and corticosterone using a Corticosterone ELISA kit (Enzo LifeScience), according to manufacturer's instructions. The balance between circulating insulin and glucose levels was determined using the homeostasis model assessment of insulin resistance (HOMA‐IR), however as non‐fasted blood samples were utilized, it can only be used to infer insulin resistance.

$$\begin{array}{c} \text{HOMA}\;\text{IR}=\frac{\left[\text{mg}\;{\text{dl}}^{‐1}\;\text{glucose}\times \mu U\;{\text{ml}}^{‐1}\;\text{insulin}\right]}{405} \end{array}$$

### 2.7 Statistical Analysis

Data were analyzed using GraphPad Prism 8 and presented as means ± SD. Normality was assessed using a Shapiro–Wilk tests with non‐normally distributed data log transformed prior to analysis. Total area under the curve was calculated using GraphPad Prisms ‘Area Under the Curve’ function and the blood glucose levels at 0, 15, 30, 60, 120, and 180 min. The effect of the WD on metabolic and behavioral markers was compared between groups (Control vs WD, *n* = 24/group) prior to the 4‐week CRS period using an independent sample *T*‐test. A two‐factor ANOVA was used to compare the impacts of time (pre‐CRS vs. post‐CRS) and the impact of diet (Control vs. WD) and stress (−CRS vs. +CRS) on metabolic, behavioral, and cardiovascular endpoints. For all two‐way ANOVA, a Sidak post hoc test was performed subsequent to identifying significant overall effects. A Pearson's correlation was performed to explore the relationship between end‐point variables (body weight, fasting plasma glucose, GTT‐AUC, and is presented as *r*(df) = *r* statistic, *p* value). Differences with a *p* < 0.05 were considered to be statistically significant.

## 3 RESULTS

### 3.1 Effects of a control and WD on body weight, glycemic control, and behavior

**FIGURE 2:**
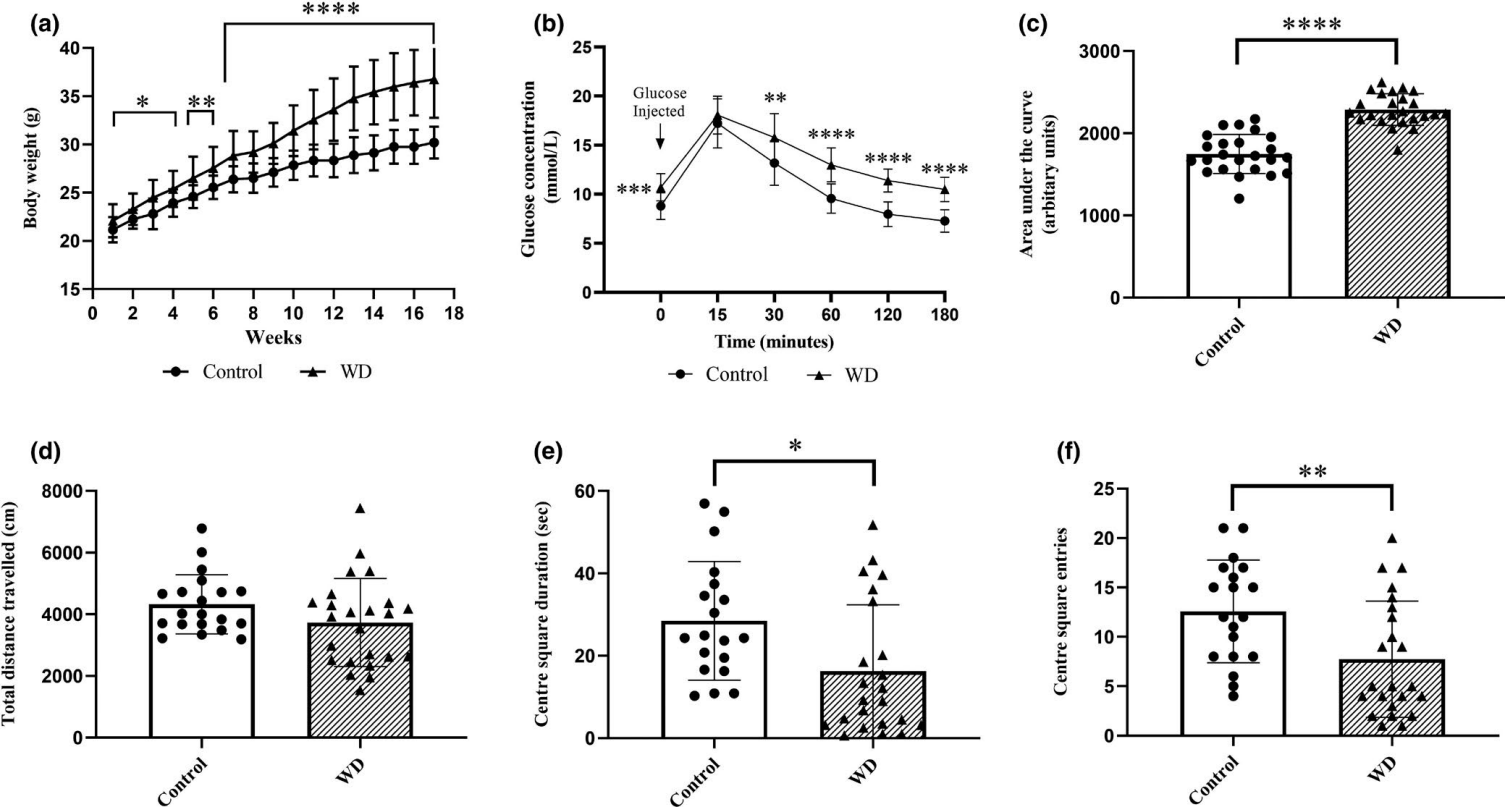
Change in body weight, glycemic control, and anxiety‐like behavior in response to dietary intervention. (a) Weekly body weights for male C57BL/6 mice on a control diet or WD for 18 weeks. (b) Glucose tolerance test results after a glucose bolus i.p. injection after a 4‐h fast. (c) Total‐AUC for the 180‐min GTT after 15 weeks of control diet or WD. Behavior was assessed using the OFT and included: (d) total distance travelled; (e) center square duration; and (f) center square entries. Data expressed as means ± SD (*n* = 24 per group). A student's *t*‐test was used for statistical and * indicates degree of significance. *Significantly different from control group (no CRS). AUC, area under the glucose curve; CRS, chronic restraint stress; GTT, glucose tolerance test; OFT, open field test; WD, western diet. *p < 0.05, **p < 0.01, ***p < 0.001, ****p < 0.0001

Both dietary groups gained body weight over the feeding period prior to CRS exposure. The rate of weight gain accelerated during weeks 8–17 in the WD group (Figure 2a). The WD worsened glycemic control, with significant elevations in fasting plasma glucose (*p* < 0.0001; Figure 2b) and GTT‐AUC (*p* < 0.0001; Figure 2c) compared to control animals. There was no difference in total distance travelled between WD and control animals prior to exposure to CRS (*p* = 0.12; Figure 2d). However, CSD (*p* = 0.014; Figure 2e) and CSE (*p* = 0.0079; Figure 2f) were significantly lower in the WD‐fed animals.

### 3.2 Effects of CRS on body weight, glycemic control, and circulating metabolic markers

**FIGURE 3:**
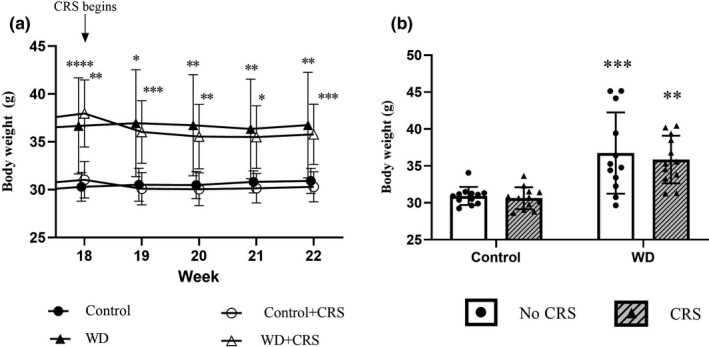
The effect of CRS on body weight in the two dietary groups. (a) Body weights assessed weekly during the CRS period (from 18 to 22 weeks of feeding) in both stressed and non‐stressed animals. (b) Endpoint body weights after the combined dietary and stress interventions. Data expressed as means ± SD (*n* = 12 per group). A two‐way ANOVA followed by a Sidak post hoc used for statistical analysis. CRS, chronic restraint stress. *Significantly different from Control group (No CRS). *p < 0.05, **p < 0.01, ***p < 0.001, ****p < 0.0001

Exposure to CRS resulted in an acute drop in body weight in both dietary groups, with the WD+CRS animals losing significantly more weight after the initial 2 weeks of CRS compared to the Control+CRS group (7% weight loss vs. 3% weight loss; *p* = 0.0004; Figure 3a). After 2 weeks of CRS, body weight stabilized in both dietary groups with no further weight gain observed in the final 2 weeks of the study, despite maintenance of WD feeding in relevant groups. Body weight at the end of the combined intervention reveals a main effect of WD feeding [Diet; *F*(1, 44) = 33.05; *p* < 0.0001; Figure 3b] with both the WD and WD+CRS groups maintaining significantly heavier body weights than the control group (*p* < 0.0006 and *p* < 0.0004, respectively). Overall, both WD groups were significantly heavier than the Control group (Table 2) with the WD causing an approximately 15% increase in body weight. This was despite the fact that several WD animals (*n* = 4) failed to gain more than 10% body weight compared to the Control group.

**TABLE 2:** Metabolic parameters at the study endpoint after the combined WD and CRS intervention

	Mean ± SD			
	Control *n* = 12	Control+CRS *n* = 12	WD *n* = 12	WD+CRS *n* = 12
Body weight (g)				
Body weight (Week 17)	30.3 ± 1.5	31.0 ± 1.90	36.7 ± 5.0***	37.9 ± 3.5****
Body weight (Week 21)	30.9 ± 1.2	30.6 ± 1.5	36.7 ± 5.5***	35.9 ± 3.2**
GTT (Week 21)				
Fasting plasma glucose (mmol/L)	7.3 ± 1.0	8.9 ± 1.1*	10.3 ± 1.4****	9.5 ± 1.4***
Total area under the curve	1614 ± 179.1	1668 ± 74.8	2179 ± 351.0****	1965 ± 169.9**
Serum (endpoint)				
Triglycerides (µM)	112.5 ± 60.2	144.9 ± 39.9	183 ± 38.2**	158.6 ± 42.5
Insulin (µU/ml)	19.2 ± 7.8	23.7 ± 9.0	54.3 ± 25.7****	46.3 ± 11.8****
Non‐fasted glucose (mmol/L)	10.9 ± 1.9	10.4 ± 1.8	12.5 ± 1.6	11.0 ± 1.0
HOMA‐IR	9.2 ± 3.9	11.1 ± 4.3	30.2 ± 4.3****	23.3 ± 6.2***
Corticosterone (pg/ml)	47.0 ± 46.7	62.3 ± 42.1	51.9 ± 28.3	49.2 ± 30.9

Note

Significantly different from Control; *p < 0.05, ** *p* < 0.01, ***p < 0.001, ****p < 0.0001.

Abbreviations: GTT, glucose tolerance test; HOMA‐IR, Homeostatic Model Assessment for Insulin Resistance.

**FIGURE 4:**
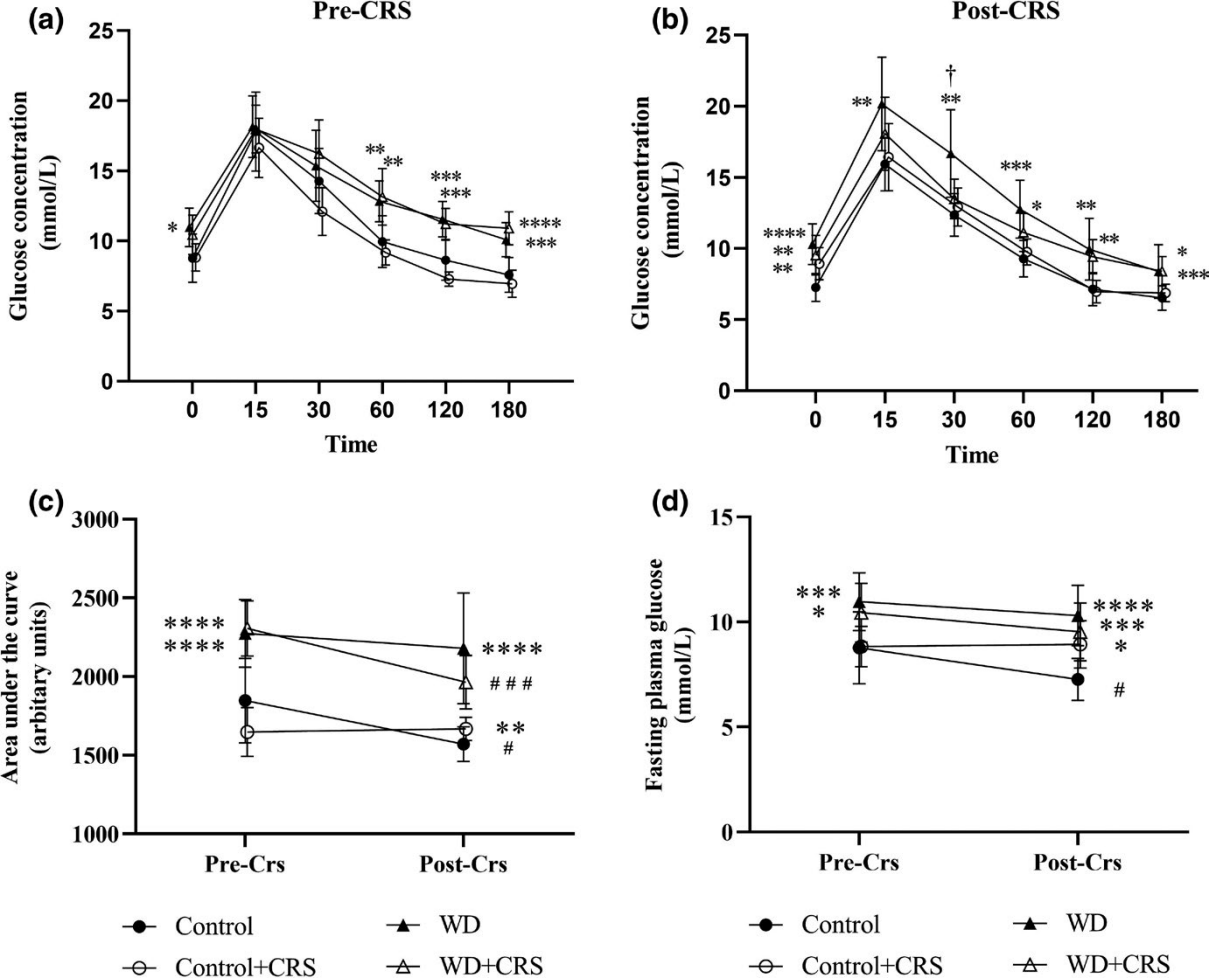
Measures of glycemic control before (pre; 15 weeks feeding) and after (post; 21 weeks feeding) the CRS period. Glucose tolerance test results after a glucose bolus i.p. injection after a 4‐h fast (a) before the stressor and (b) after the stressor. (c) Fasting plasma glucose prior to and after the CRS period. (d) GTT AUC prior to and after the CRS period. A two‐way ANOVA followed by a Sidak post hoc was used for statistical analysis. Significantly different from control group (no CRS). AUC, area under the glucose curve; CRS, chronic restraint stress; GTT, glucose tolerance test; OFT, open field test; WD, western diet. *p < 0.05, **p < 0.01, ***p < 0.001, ****p < 0.0001. Significantly different from previous time point. ^#^ *p* < 0.05, ^##^ *p* < 0.01, ^###^ *p* < 0.001. WD significantly different from WD+CRS. ^†^ *p* < 0.05

Glucose tolerance curves reveal minor improvements to glycemic handling within the WD+CRS group compared to the WD group 30‐min after the glucose bolus injection (Figure 4b; *p* = 0.02). This resulted in a significant 14% reduction of GTT‐AUC in the WD+CRS group over the CRS period (Figure 4c; *p* = 0.0006). Interestingly, a similar reduction in GTT‐AUC was noted with the control group (*p* = 0.01), but not within the Control+CRS group or WD group (Figure 4c *)*. A generalized decrease in fasting plasma glucose concentrations was noted over the CRS period [Time; *F*(1, 42) = 8.24; *p* = 0.006; Figure 4d].

At the study endpoint, fasting plasma glucose levels were affected by both interventions [Diet × CRS; *F*(1, 42) = 10.80; *p* = 0.0021; Table 2) with significantly higher levels observed in Control+CRS group (*p* = 0.02), the WD group (*p* < 0.0001), and WD+CRS group (*p* = 0.0006) compared to the Control group. Similarly, WD feeding was also associated with a higher AUC [Diet: F (1, 42) = 48.01; *p* < 0.0001; Table 2] with significantly higher levels noted in both the WD and WD+CRS groups compared to the Control group (*p* < 0.0001 and *p* = 0.0025, respectively). A minor interaction with both interventions was noted for GTT‐AUC [Diet × CRS: *F*(1, 42) = 4.24; *p* = 0.05] with a trend for improved glucose tolerance noted within WD+CRS group compared to the WD group (*p* = 0.09; Table 2).

**FIGURE 5:**
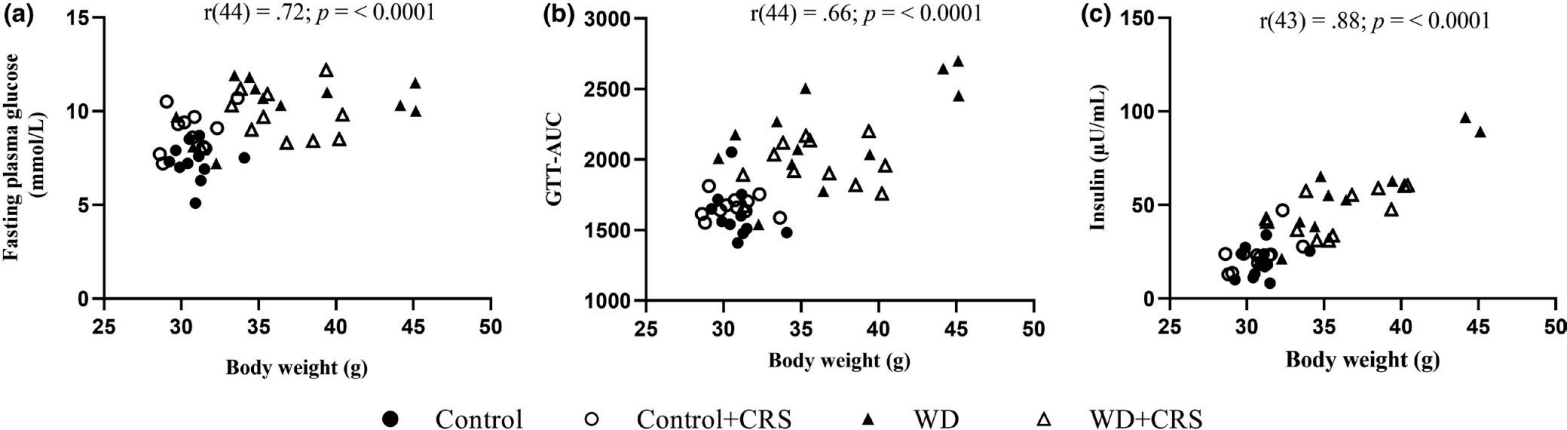
Correlation analysis after the CRS describing the relationship between body weight and (a) Fasting plasma glucose, (b) Total area under the curve of the GTT, and (c) circulating insulin in non‐fasted animals. A Pearson's correlation was used to determine statistical correlations between variables. CRS, chronic restraint stress; GTT, glucose tolerance test

Blood triglyceride levels were significantly increased by WD feeding [Diet: *F*(1, 35) = 8.39; *p* < 0.01], with post hoc analysis revealing the WD group had significantly higher triglyceride levels when compared to the Control group (*p* = 0.0094; Table 2). Circulating triglyceride levels were not altered in CRS animals compared to their respective dietary controls, however, a trend for an interactive effect was observed [Diet × CRS: *F*(1, 35) = 3.80; *p* = 0.06]. Blood insulin was significantly elevated by WD feeding [Diet: *F*(1, 41) = 49.95; *p* < 0.0001; Table 2], with elevated levels observed in both WD and WD+CRS groups when compared to Control (*p* < 0.0001 and *p* < 0.0001, respectively). The WD had a modest effect on non‐fasting glucose [Diet: *F*(1, 41) = 4.93; *p* = 0.02; Table 2) with significantly higher levels observed in the WD group compared to the Control+CRS group (*p* = 0.02), but not the Control group (*p* = 0.07). A similar pattern of change to insulin was noted in HOMA‐IR with a strong effect of WD‐feeding [Diet: *F*(1, 40) = 45.5; *p* < 0.0001; Table 2]. No significant differences in baseline corticosterone concentrations were observed between dietary groups or in response to CRS (Table 2). Correlation analysis revealed a strong relationship between body weight and fasting plasma glucose (Figure 5a), GTT‐AUC (Figure 5b), and insulin concentrations (Figure 5c).

### 3.3 Effect of CRS on animal behavior

**FIGURE 6:**
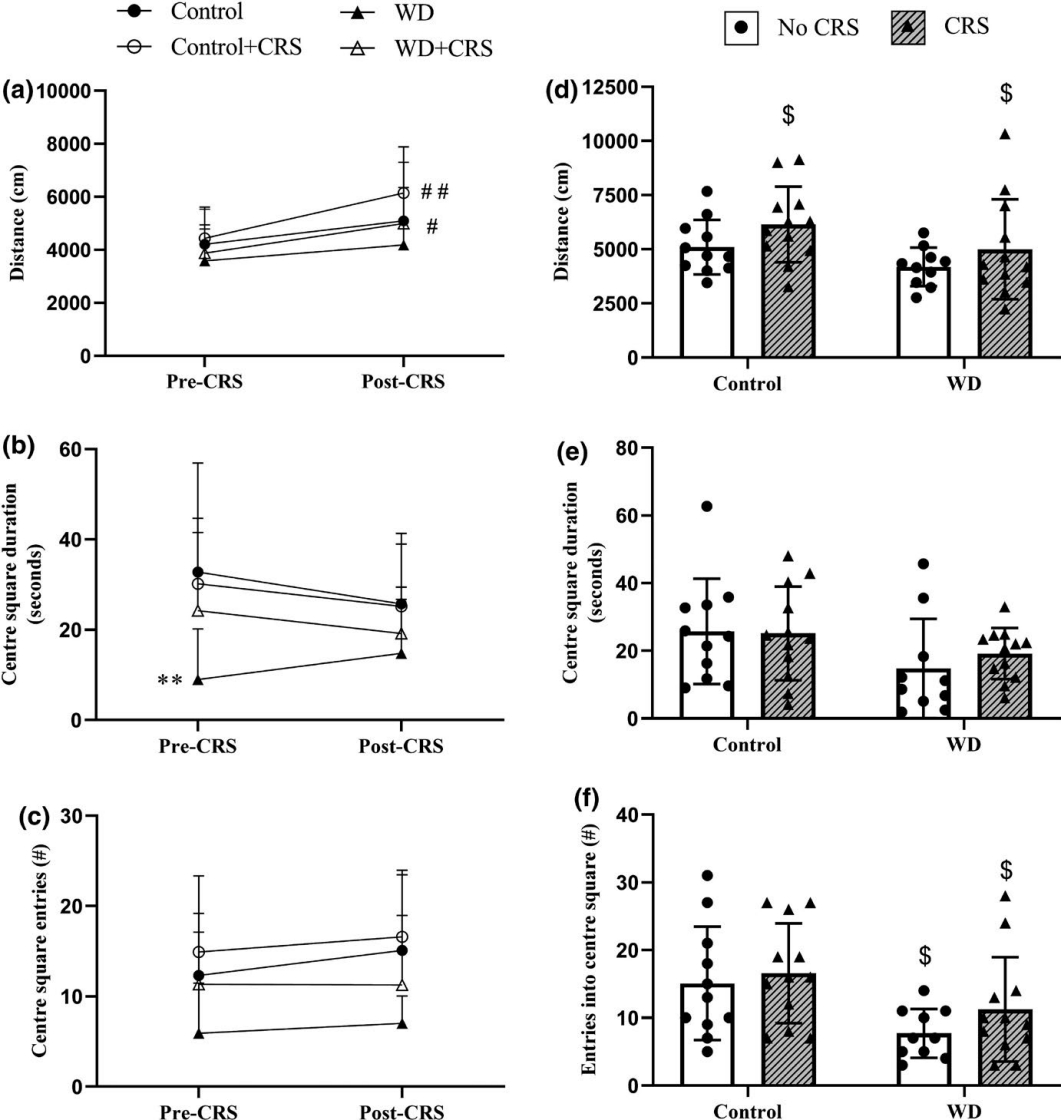
Open‐field behavior before (pre; 17 weeks of feeding) and after (post; 21 weeks of feeding) the chronic restraint stress (CRS) period in both stressed and un‐stressed animals. (a) Total distance travelled; (b) center square duration; (c) center square entries. In addition, the same behavioral parameters are shown for each group at the end of the experimental protocol, including: (d) total distance travelled; (e) center square duration; (f) C center square entries. Data expressed as means ± SD (*n* = 10–12). A two‐way ANOVA followed by a Sidak post hoc was used for statistical analysis. $ represents a significant main effect of the intervention. * and ^#^ indicate significant post hoc differences. Different from Control group (No CRS). **p < 0.01. ^#^Different from previous time point. ^#^ *p* < 0.05, ^##^ *p* < 0.01

Some aspects of behavior changed with CRS. Notably, we saw an increase in total distance travelled after the two dietary groups were subjected to the CRS period [Time: F(1,38)=23.80; *p*<0.0001; Figure 6a]. This was most notable for the Control+CRS (*p* = 0.004) and WD+CRS group (*p* = 0.03). Both CSD [Group effect: *F*(3, 43) = 3.90; *p* = 0.015; Figure 6b] and CSE [Group effect: *F*(3, 43) = 4.40; *p* = 0.023; Figure 6c] were significantly lower in the WD animals, but significant changes in response to CRS were not observed for either control diet or WD animals.

At the study endpoint nonsignificant reductions in total distance travelled among WD animals was still evident [Diet: *F*(1, 41) = 3.84; *p* = 0.056; Figure 6d]. In contrast, CRS groups were associated higher total distance travelled [CRS: *F*(1, 41) = 3.66; *p* = 0.06; Figure 6d]. Similarly, CSD tended to be lower [Diet: *F*(1, 38) = 3.42; *p* = 0.07; Figure 6e] and CSE were significantly lower [Diet: *F*(1, 41) = 6.76; *p* = 0.01; Figure 6f] in WD animals. Post hoc reveals that exposure to CRS did not result in significant differences in behavioral measures when compared to their respective dietary controls.

### 3.4 Effect of a WD diet and CRS on myocardial function and tolerance to I/R

Baseline cardiac function prior to I/R, and functional recovery after I/R, was assessed using a Langendorff heart perfusion model. As expected, cardiometabolic risk factor values for the subgroup of animals that was randomly selected for heart perfusions mirrored those documented for the four larger groups of mice used in the whole study (Table 2; Table S1) . Baseline cardiac function was comparable between groups (Table 3). Significantly higher heart weights were noted in both the WD and WD+CRS groups [Diet: *F*(1, 25) = 9.83; *p* = 0.00] compared to the control (~20% vs. control), however, the heart‐to‐body ratio was unaffected by either the WD or CRS intervention.

**TABLE 3:** Baseline cardiovascular function in Langendorff perfused hearts

	Mean ± SD (*n* = 5–8 per group)			
	Control *n* = 5	Control + CRS *n* = 8	WD *n* = 8	WD + CRS *n* = 8
Heart weight (g)	161 ± 29.9	182.5 ± 21.6	204.5 ± 31*	196.9 ± 20.1*
Heart‐to‐body weight ratio	5.2 ± 1.0	6.0 ± 0.5	5.5 ± 0.9	5.40 ± 0.4
Coronary flow (ml min^−1^)	4.2 ± 2.1	3.3 ± 1.1	4.0 ± 1.2	5.0 ± 1.7
Diastolic pressure (mmHg)	3 ± 3	5 ± 4	4 ± 5	6 ± 1
Systolic pressure (mmHg)	120 ± 20	116 ± 24	107 ± 25	124 ± 15
Developed pressure (mmHg)	118 ± 22	111 ± 26	103 ± 27	117 ± 15

Note

Significantly different from Control (No CRS). *p < 0.05.

**FIGURE 7:**
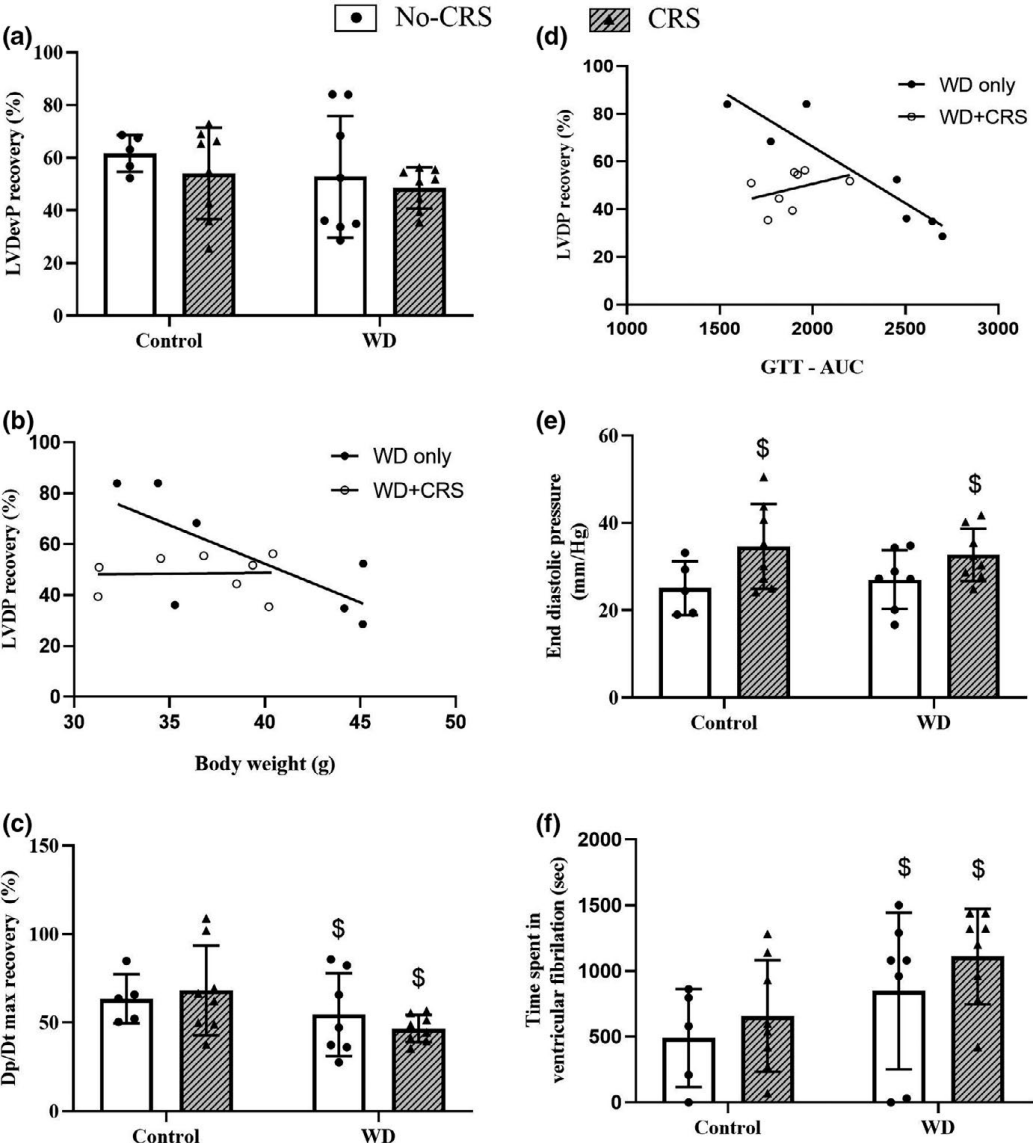
Postischemic functional outcomes in Langendorff perfused hearts. (a) Recovery of LVDevP; (b) relationship between LVDevP and GTT area under the curve in WD mice only (±CRS); (c) relationship between LVDevP and endpoint body weight in WD mice only (±CRS); (d) recovery of left ventricular diastolic pressure; (e) recovery of +dP/dt max; (f) total time in ventricular fibrillation throughout reperfusion. Recovery values were determined 45 min into reperfusion. Data expressed as means ± SD (*n* = 5–8). A two‐way ANOVA followed by a Sidak post hoc was used for statistical analysis. CRS, chronic restraint stress; GTT, glucose tolerance test; LVDevP, left ventricular developed pressure; WD, western diet. $ represents a significant main effect of the intervention. A linear regression was used to determine the statistical relationship between LVDevP recovery and GTT‐AUC and body weight in WD groups

Functional recovery was assessed 45‐min after reperfusion. Reperfusion left ventricular developed pressure (LVDevP) recovery was not affected by the WD nor CRS intervention when compared with the Control group (Figure 7a). This lack of an effect with the WD may have been due to the inclusion of several low weight nonresponsive mice in the WD group. This lack of responsiveness to the obesogenic diet is highlighted in Figure 7b,c with low‐weight, glucose tolerant WD mice showing no change in LVDevP recovery and high weight, glucose insensitive mice showing poor LVDevP recovery. Interestingly, although a strong inverse relationship between LVDevP recovery and GTT‐AUC was observed in the WD mice (Figure 7b *)*, there was no such relationship observed in WD+CRS mice for LVDevP recovery and GTT‐AUC (Figure 7b) or body weight (Figure 7c). End diastolic pressure during reperfusion was significantly higher in animals subjected to CRS [Stress: *F*(1, 25) = 7.74; *p* = 0.01; Figure 7d], however post hoc reveals no group differences. Furthermore, *dP*/*dT* max recovery was significantly lower in the WD animals [Diet: *F*(1, 25) = 5.12; *p* = 0.05; Figure 7e]. Time spent in postischemic ventricular fibrillation was significantly higher in WD animals [Diet: *F*(1, 25) = 5.01; *p* = 0.03; Figure 7f], and a nonsignificant increase for greater time spent in ventricular fibrillation was noted in the WD+CRS compared to the Control group (*p* = 0.09). No significant additive effects of WD and CRS were noted in the functional indices assessed.

## 4 DISCUSSION

This study investigated the effects of a WD and CRS, on behavior, cardiometabolic risk factors and myocardial response to I/R in a murine model. Our data indicates WD+CRS mice exhibit improvements in glucose tolerance while increasing myocardial susceptibility to I/R injury. Primary findings of this study are: (1) the WD promotes obesity and glucose intolerance; (2) CRS improved body weight and body weight gain in both control and WD mice; (3) CRS improved glucose tolerance in WD‐fed animals with no alterations to insulin levels; (4) the WD induced robust changes in exploratory behavior which was lost when the WD was combined with CRS; (5) despite WD+CRS mice exhibiting a more favorable cardiometabolic risk profile than WD‐fed animals, this did not translate into improvements in postischemic myocardial outcomes.

### 4.1 Effect of a WD and CRS on cardiometabolic risk factors

In the current study, the WD‐induced DIO with metabolic dysregulation, evidenced by increased body weight, insulin insensitivity, hyperlipidemia, and hyperinsulinemia. However, several mice in this group were non‐responders and failed to become obese. These animals only displayed moderate increases in body weight and cardiometabolic risk markers. This phenomenon of responders and non‐responders to obesogenic diets has been characterized in the literature (Dourmashkin et al., 2006; Tulipano et al., 2004) and limits our ability to appropriately interpret the effects of obesity on cardiometabolic and behavioral outcomes.

Accumulating evidence highlights the potential for crosstalk and synergistic interactions between obesogenic feeding and chronic stress in the development of disease (Kelly & Ismail, 2015; Ortiz & Sapunar, 2018; Ulrich‐Lai et al., 2015). Contrary to this, there is also evidence that palatable diets may be beneficial in models of stress (Fleur et al., 2005; Maniam & Morris, 2010), and that stress can ameliorate the metabolic dysregulation observed in DIO models (Bates et al., 2007; Kai et al., 2000). The current study found that the introduction of CRS caused an initial drop in body weight in control and WD animals, with a sustained slowing of weight gain during the 4 weeks of CRS exposure. These observations are consistent with the literature with uncontrollable stress inducing an anorexigenic phenotype in rodent models, resulting in weight loss (Bruder‐Nascimento et al., 2013; Fu et al., 2009; Patterson & Abizaid, 2013). This study also noted that CRS‐induced moderate changes to glycemic control which appeared to be dependent on the diet. Control‐fed mice showed evidence of hyperglycemia in response to CRS which has been reported previously in similar models (Zardooz et al., 2006; Zheng et al., 2018). Interestingly, non‐stressed control mice showed significant improvements in fasting plasma glucose and glycemic control during the same period resulting in elevated levels of fasting glucose in the Control+CRS group at the experimental endpoint. The reasons for this improvement in the control group is unclear but may be related to habituation to handling and the repeated GTT procedure resulting in a loss of stress‐induced hyperglycemia prior to assessment (Bates et al., 2008; Ghosal et al., 2015; Girotti et al., 2006).

Results from the present study also suggest that CRS may alter metabolic dysregulation in DIO by improving body weight and glycemic control. Food consumption was not assessed in the current study, however the improved metabolic control is likely the result of CRS‐induced appetite suppression and/or an increased metabolic rate and consequent weight reduction in the WD animals (Rabasa et al., 2011). Interestingly, while a reduction in food intake and body weight may have been a confounding factor to improvements in metabolic parameters, WD+CRS exhibited marked improvements to glycemic tolerance (~15%) with minimal changes in body weight (~5%). Indeed, at the study endpoint, relatively small (~2.4%) differences in the body weights of the WD and WD+CRS animals resulted in more pronounced differences in glucose tolerance (~10%). The current data does not suggest a synergistic adverse effect of a WD and CRS on glucose homeostasis, but instead supports a mild protective effect in WD‐fed animals with impaired glucose tolerance similar to that documented by Packard et al. (2014).

This finding is contrary to several studies (Du Toit et al., 2020; Fu et al., 2009; Pereira et al., 2016; Sousa Rodrigues et al., 2017), but corroborates several others (Bates et al., 2008; Bruder‐Nascimento et al., 2013; Finger et al., 2012; Garcia‐Diaz et al., 2007; Kai et al., 2000; Packard et al., 2014), and highlights the general lack of consistency in the effects of chronic stress on metabolic dysregulation. Habituation to the CRS and the loss of stress‐induced hyperglycemia may also explain the findings observed in the current study (Grissom & Bhatnagar, 2009; Rabasa et al., 2015). Habituation to the CRS after the initial drop in body weight is supported by the absence of robust changes to corticosterone levels measured at study endpoint and our lack of changes to insulin in CRS‐exposed groups which contrasts our previous work (Du Toit et al., 2020; Grissom & Bhatnagar, 2009). Furthermore, the strong association between body weight and circulating insulin levels seen in the control and diet groups was unchanged by the exposure of these groups to CRS which may suggest that the stressor was no longer impacting systemic metabolism and physiology. Taken together, varied outcomes when assessing DIO and stress may reflect the temporal characteristics of stress‐induced metabolic regulation or the impact that different stressors play in the stress‐response (Cavigelli et al., 2018; Finger et al., 2012; Zardooz et al., 2006). The relevance of these stress‐induced metabolic differences on cardiovascular outcomes is still unknown, however it raises questions regarding the efficacy of traditional metabolic risk factors for cardiovascular disease (CVD) within the context of an active stressor that is metabolically disruptive or a habituated stressor.

### 4.2 Effect of a WD and CRS on locomotion and exploratory behavior

DIO has been shown to influence cognition and induce anxiety‐like behavior (Andre et al., 2014; Pini et al., 2017), however mechanisms underpinning these effects are incompletely characterized. In the current study, the WD had no effect on total locomotion, consistent with prior studies (Garcia‐Diaz et al., 2007; Santos et al., 2018). A modest decrease in CSD and CSE was observed in response to the WD, indicative of anxiety when confronted with the open‐field arena (Prut & Belzung, 2003). This support prior evidence that high‐fat and high‐sucrose diets promote anxiety‐like behavior in rodents (Eudave et al., 2018; Rebolledo‐Solleiro et al., 2017). All groups showed an increase in locomotion in the OFT conducted after the 4‐weeks of CRS. This improvement may again be a consequence of habituation to the experimental testing arena resulting in a generalized increase in locomotion in the novel open field environment. CRS mice showed a greater increase in locomotion during this period. Mild stressors have been shown to promote hyperactivity in the OFT (Garcia‐Diaz et al., 2007; Zimprich et al., 2014), whereas social and severe stressors induce a loss of locomotor activity (Bowman et al., 2002; Guedri et al., 2017). Despite this stress‐induced hyperactivity, exploratory behavior was unaltered by CRS in either dietary group, suggesting that the current homotypic stressor protocol (restraint stress) is insufficient to induce overt behavioral changes typical of chronic stress‐induced anxiety. Additional behavioral assessments of learned helplessness, learning and memory, and social activity may have revealed more subtle long‐term alterations to cognition and behavior induced by the CRS (Huang et al., 2015; Wood et al., 2008; Zain et al., 2018).

### 4.3 Effect of a WD and CRS on myocardial tolerance to I/R

Myocardial tolerance to an I/R insult is highly dependent on normal metabolism to ensure cell survival. Insulin resistance in murine obesity is associated with detrimental changes to the cardioprotective reperfusion injury salvage kinase pathway activated in response to a I/R insult (Donner et al., 2013; Poncelas et al., 2015). In support of this, the current study noted that LVDevP recovery was inversely correlated with body weight and glucose tolerance. A limitation of the current study was that a subset of mice used in the Langendorff perfusion experiments failed to respond to the WD‐feeding and did not present with pronounced obesity or metabolic dysregulation. This subset of animals increased the variability of heart perfusion data of the WD‐fed animals. The absence of statistically significant differences in the cardiac outcomes between groups contrasts with our previous work (Donner et al., 2013; Du Toit et al., 2020; Toit et al., 2008) and is likely due to the sample size and number of animals in our WD groups that were non‐responders to the obesogenic diet. Despite this variability in cardiac outcomes, the study did observe more prolonged reperfusion fibrillation in the WD‐fed animals which is consistent with evidence showing that metabolic diseases promote arrhythmogenesis (Albarado‐Ibanez et al., 2013). Similarly, while no significant dietary effect was noted for LVDevP recovery, dP/dt max recovery was reduced in WD groups and supports a loss of pressure recovery in DIO animals. The heart‐body‐weight ratio was unchanged in WD groups suggesting that the increase in heart weight is due to the greater body weight rather than hypertrophy. No additive effect of CRS was observed in these two‐perfusion metrics.

Interestingly, CRS did not induce significant changes in LVDevP recovery on its own or in combination with WD feeding. This was unexpected since our previous work demonstrated a detrimental synergistic effect of these two interventions (Du Toit et al., 2020). Two considerations may explain this difference. The first is that the current study utilized a longer CRS protocol than our previous work which facilitated a more marked habituation to the stressor and a reduction in the physiological response (Grissom & Bhatnagar, 2009). Second, the current study did not obverse synergistic disruption to metabolic homeostasis in the combined intervention group which resulted in comparable insulin levels between WD groups and evidence of improved glycemic handling in WD+CRS groups. Both factors could have resulted in the lack of changes to reperfusion LVDevP in WD+CRS. However, one would expect that there would still be alterations to LVDevP recovery in the WD+CRS group as a result of stress‐induced injury and remodeling that occurred prior to habituation (Manukhina et al., 2021; Zhang et al., 2019). Despite this, there remained evidence of postischemic contractile dysfunction in the hearts of mice exposed to CRS as indicated by the elevation in reperfusion EDP. Furthermore, there was evidence of disruption to the relationship between metabolic and postischemic functional outcomes in mice exposed to WD+CRS compared to WD only mice. This is most likely due to functional recovery being affected by additional stress pathways (autonomic nervous system, inflammatory signaling, and oxidative stress) activated by CRS (Adameova et al., 2009; Dorn et al., 1997; Mercanoglu et al., 2008).

Although CVD is strongly associated with both metabolic and stress‐related mood disorders, few studies assess the interactive effects of these co‐morbidities on the heart and its response to ischemic insult (Mottillo et al., 2010; Nicholson et al., 2006). Our understanding of this relationship is further complicated by mode of stress induction (frequency, predictability, and type) that can result in diverse metabolic and cardiac outcomes (Crestani, 2016; Patterson & Abizaid, 2013). This raises questions about the relationship between metabolic disturbances and CVD outcomes under different physiological stress states (i.e., active vs. habituated). In our previous work, we noted that a short stressor (30 mins/day for 3 weeks) after a 10‐week WD diet feeding program synergistically disrupted metabolism culminating in worsened ischemic tolerance (Du Toit et al., 2020). The current study did not observe synergistic effects on metabolism, but still noted limited myocardial dysfunction in CRS‐exposed mice likely due to effects not related to metabolism. Taken together, this supports a role for stress‐induced insulin resistance to worsen ischemic tolerance in DIO, however, there may be additional mechanisms contributing to diverse outcomes. Agrimi et al., noted significant reductions to echocardiography‐derived ejection fraction and fractional shortening in obese C57BL/6J mice exposed to a chronic stressor (Agrimi et al., 2019). The authors also noted evidence of elevated myocardial fibrosis and apoptosis and attribute the changes to the synergistic depletion of brain‐derived neurotrophic factor signaling. Unfortunately, the authors did not assess corticosterone or markers of metabolism which makes it difficult to ascertain the degree of stress activity or whether this loss of neurotrophic signaling occurred concurrently with dysregulated metabolism. Within a clinical setting, the temporal dynamics (i.e., active, habituated, maladaptive) and characteristics (frequency, predictability, and type) of a stressor may result in variable cardiometabolic profiles in individuals with obesity that may not be accurate representations of current and future CVD and ischemic intolerance risk. Further research is warranted into the temporal relationship between metabolism and myocardial ischemic risk in multimorbidities to further our understanding of CVD risk.

### 4.4 Study limitations

Several limitations and constraints impacted our findings that are worth noting. The presence of low‐weight WD‐fed mice and CRS mice that possibly habituated to the stressor limited the robustness of the model and our findings. While we were still able to investigate the interactive effects of both interventions on cardiometabolic and behavioral parameters, the heterogeneity and modest changes to study outcomes hindered our ability to investigate potential mechanistic pathways of interest. Furthermore, while the OFT is an established tool for the assessment of anxiety‐like behavior in mice, we were unable to fully characterize the behavioral phenotype of WD and CRS mice to determine whether changes occurred to additional behavioral and cognitive indices such as learned helplessness, learning and memory, motor coordination, or social interactions. The current study was unable to assess the combined effects of WD and CRS on fasting metabolic markers which may have provided more information regarding the basal metabolic state of these animals Moreover, use of an ex vivo Langendorff model to assess myocardial ischemic tolerance may have mitigated the impact of systemic and neural pathways on functional recoveries. Lastly, there is strong evidence to suggest sex‐specific differences in both the stress‐response and cardiovascular outcomes (Bangasser & Wiersielis, 2018; Möller‐Leimkühler, 2007). We are therefore unable to determine if female mice would exhibit the same stress‐induced changes to metabolic and myocardial ischemic parameters with varying stress paradigms. With these limitations in mind, future research should investigate the potential interactions between chronic stress and DIO on the relationship between metabolism and myocardial function to determine mechanisms of action and the risk profile of varying stress‐phenotypes (i.e., active stressor vs. habituated; physical vs. psychological).

## 5 CONCLUSION

In summary, mild CRS improved body weight and glucose tolerance in the WD animals but had no effect on these measures in control diet‐fed animals. Despite improvements to body weight and glucose tolerance, CRS did not improve myocardial tolerance to I/R and selectively worsened postischemic contractility. The combination of WD+CRS did not elicit the typical association between glucose intolerance and exacerbated ischemic injury. This highlights a potential limitation of metabolic markers in determining CVD risk in cases of mild chronic stress and obesity. Furthermore, while mild stressors may be clinically relevant, application of chronic stressors to which the animals do not habituate (such as heterotypic stressors) may be necessary to reveal potential synergistic effects between DIO and stress‐related disorders (Zhu et al., 2014). Further research is required to delimitate and characterize the temporal relationship between the stress‐response and cardiometabolic parameters. We also need to determine what the relative contributions of metabolic dysregulation and stress‐induced neuro‐endocrine overactivity and inflammation are to CVD progression and ischemic intolerance in multimorbid conditions. Although this study has its limitations, it provides evidence for potential interactions between chronic psychological stress and a WD in modulating CVD outcomes and highlights the complexities of the stress‐response that warrant further research.

## CONFLICT OF INTEREST

The authors declare that they have no competing interests.

## AUTHOR CONTRIBUTIONS

*Conceptualization of study design*: Amanda J. Cox, Jason N. Peart, John P. Headrick, and Eugene F. du Toit. *Data acquisition*: Jason N. Peart, John P. Headrick, and Kyle Hatton‐Jones. *Data acquisition, analysis, and interpretation*: Kyle Hatton‐Jones, Eugene F. du Toit, and Amanda J. Cox. *Writing‐original draft preparation*: Kyle Hatton‐Jones. *Writing‐review, editing, and supervision*: Amanda J. Cox and Eugene F. du Toit. All authors have read and approved the final version of this manuscript and agree to be accountable for all aspects of the work in ensuring that questions related to the accuracy or integrity of any part of the work are appropriately investigated and resolved. All persons designated as authors qualify for authorship, and all those who quality for authorship are listed.
